# Conceptualisations of the social determinants of health among first‐year dental students

**DOI:** 10.1186/s12909-021-02602-1

**Published:** 2021-03-17

**Authors:** Alexander C L Holden, Delyse Leadbeatter

**Affiliations:** 1 The University of Sydney School of Dentistry, Faculty of Medicine and Health, 2–18 Chalmers Street, NSW 2010 Surry Hills, Australia; 2 The University of Sydney School of Public Health, Faculty of Medicine and Health, Camperdown, Australia

**Keywords:** Social Determinants of Health, Oral health, Dental education, Professionalism, Structural competency, Dental Public Health, Curriculum

## Abstract

**Background:**

Social conditions have a significant impact on the health of individuals and populations. While the dental curriculum is focused on teaching students about the diseases that affect the dentition and oral structures from a biomedical perspective, education about the social determinants of health is frequently regarded as less important. Thus, it occupies a smaller and disconnected part of the dental curriculum. The aim of this study was to explore the ways dental students conceptualised the social determinants of health after one year in dental school.

**Methods:**

Reflective statements written by first year dental students at the end of the first year of study were collected. This qualitative study has an interpretivist basis and a thematic analysis of the reflections was conducted by two researchers. Metzl’s structural competencies were used as a further analytic device.

**Results:**

Four inter-related themes were identified: First, professional attitudes taken up by students influence their conceptions. Second, structural barriers to students understanding social determinants of health generate partial understandings. Thirdly, the social gulf that exists between the student body and people of different circumstances provides context to understanding the student’s perspectives. Finally, we described how students were learning about the social determinants of health over the academic year.

**Conclusions:**

Dental students face several challenges when learning about the social determinants of health, and translating these learnings into actions is perhaps even more challenging. Metzl’s structural competencies provide a framework for advancing students’ understandings. One of the most important findings of this research study is that coming to an understanding of the social determinants of health requires sustained attention to social theories, practical experiences as well as institutionalised attitudes that could be achieved through an intentional curriculum design.

## Introduction

Understanding the social determinants of health and structural inequality is a key competency for all dental students to develop so they can provide socially competent care, especially for patients from groups who shoulder the greatest burden of disease due to their socioeconomic status and vulnerability within society. Despite this, non-technical subjects within the dental curriculum tend to struggle against the dominance of clinical and practical subject areas [1], with learning about the biopsychosocial elements of dentistry being heavily influenced by the hidden curriculum [2]. While it may be accepted that teaching and learning about the social determinants of health are important, the dental profession as a collective has often failed to take decisive action against inequality and inequity in oral health. Professional narratives often take a neo-liberal approach (for example, market-oriented reforms such as eliminating cost barriers that minimize access to health care services) to oral health injustice which focuses upon the importance of personal responsibility for lifestyle choices and the affordability of care [3, 4]. To ensure that the dental profession remains ‘fit-for-purpose’ into the 21st Century, having an immutable voice in advocating for global reductions in oral health disparities [5], dental curricula need to correct their focus; incorporating the social determinants of health as a theme running through every learning activity. Dental schools must ensure that students not only understand how the social determinants of health impact the mouth and oral wellbeing, but also develop a deep sense of social responsibility and capacity to act towards their amelioration and management.

Dental curricula teach students about the social and structural determinants of health through a variety of different approaches, with traditional activities such as lectures and tutorials being provided alongside activities embedded within the community, where students undertake service-learning placements. While deep engagement of dental students with communities is in evidence in some reports of service-learning [6, 7], Furlini et al. [8] noted a generalised lack of community engagement in community-based placements in dental education. A lack of co-creation and community consultation may be indicative of activities being driven by paternalistic values, rather than community self-determination in the development of health services being seen as an intrinsic component of dental education. Lévesque et al. [9, 10] reported on a collaboration between academics, individuals experiencing poverty, a charitable organisation and key stakeholders from the dental profession to develop an educational film on poverty. Lévesque and Bedos [11] noted that curricula in dental schools should challenge negative stereotypes and judgements about those experiencing financial and social hardship, with other research finding that junior dental students were aware of their professional responsibility to care for the underserved, but were unsure of their role in this duty [12]. The importance of curricula actively addressing how dental students encounter and conceptualise the social determinants of health is highlighted by Loignon et al. [13] who found that there was a need to challenge attitudes in dentists that poverty was caused by a lack of personal responsibility. Foster Page et al. [14] found that dental students may not identify themselves as agents of change, tacitly accepting that dentistry is driven by market values.

This research focuses upon how the first-year curriculum within the four-year, postgraduate Doctor of Dental Medicine (DMD) program at the University of Sydney School of Dentistry helps students on the course to begin to conceptualise the social determinants of health and their relevance to dentistry. Presently, students participate in a series of lectures and workshops that explore how the social determinants of health impact the lives of patients and impact oral health outcomes. Of particular note is a workshop where a social enterprise external to the university, called ‘The Big Issue’, is invited to present to the students on their work which provides individuals who are homeless or at risk of homelessness the opportunity to earn an income through selling ‘The Big Issue’ magazine. The Big Issue attends the session with an employee of the charity and one of the magazine sellers who has been given training and support to share their narrative on their life story. The sellers who speak, detail for the students the hardships they have faced and how they manage the deprivation that many of the magazine sellers have faced, with many continuing to face daily challenges relating to their social and financial situations.

This research explores how the learning experiences within the first year of the DMD course have impacted upon students’ conceptualisation of the social determinants of health. This is achieved through the analysis of written reflections completed as part of the course where students discussed their understanding of the social determinants of health. The research study is based in the interpretivist paradigm, in that it accepts that the social world is understood by those participating in it, in this instance, the first-year dental students [15]. Thus, individual accounts are taken on face-value and researchers aim to illuminate structural and social forces and explain actions based on insights given by participant’s perspectives. Metzl and Hanson [16] discuss the concept of structural inequity within healthcare:

“Increasingly, we hear that low-income African Americans are unable to comply with doctors’ orders to take their medications with food, not because they harbor cultural mistrust of the medical establishment, but because they live in food deserts with no access to grocery stores. Or, that Central American immigrants who are at risk for Type-II Diabetes refuse to exercise, not because they are uneducated about the benefits of weight reduction, but because their neighborhoods have no gyms or sidewalks or parks.” (page 127).

Structure is therefore a complex construct, being comprised of physical components (such as living environments and resources), political components (such as legislation and public policy) and social components (such as racism), all of which interact and synergise with one another in complex ways that may impact health negatively.

In their theoretical framework of structural competency which we have adopted and applied in this research, Metzl and Hanson [16] outline five core competencies to develop healthcare students’ ability to manage structural inequalities in their future practice. These competencies transcend the individual clinician: patient interaction to consider the broader structural influences upon health. The five competencies are: (1) recognising the structures that shape clinical interactions (developing students’ understanding of how economic, physical, and socio-political forces may influence healthcare decisions); (2) developing an extra-clinical language of structure (recognising and addressing the lack of developed narrative and consideration of environments in contrast to the plethora of discussion relating to biomedicine and the biological effects of lived environments); (3) rearticulating “cultural” formulations in structural terms (encouraging focus upon structural elements (such as policy or institutions) rather than solely focusing upon cultural explanations for health disparities); (4) observing and imagining structural interventions (identifying financial, legislative or cultural decisions that lead to structural inequities and these might be addressed through socio-political change); and (5) developing structural humility (recognising where the limits of structural competency lie for health practitioners). These competencies are helpful for considering how students’ learning may be oriented towards considering the social determinants of health beyond the immediacy of the dental surgery and will be used as the theoretical framework within this analysis.

## Methods

As part of the first year of the Doctor of Dental Medicine course at the University of Sydney, students are asked to complete a reflective task, of no less than 500 words: “Based on your experiences in DMD 1 and drawing on your life experiences, write a reflective piece about your understanding of the social determinants of general and oral health.” Students’ written accounts were submitted de-identified online through Turnitin and feedback was given by one of the authors (ACLH) to prompt further reflection. In total, 92 students, the entirety of the year, took part in this compulsory assessment task.

Ethics approval was given by the University of Sydney Human Research Ethics Committee (Project number: 2018/520) for students to be approached to gain consent for the inclusion of their reflections within this study. To avoid coercion, the researchers did not engage directly with the students to gain consent with this process being managed at arms-length by the professional staff team. All students were provided with participant information statements and written consent to participate was gained from students who were willing for their reflections to be incorporated into this study for analysis. The data were collected from the students in late 2018.

**Fig. 1:**
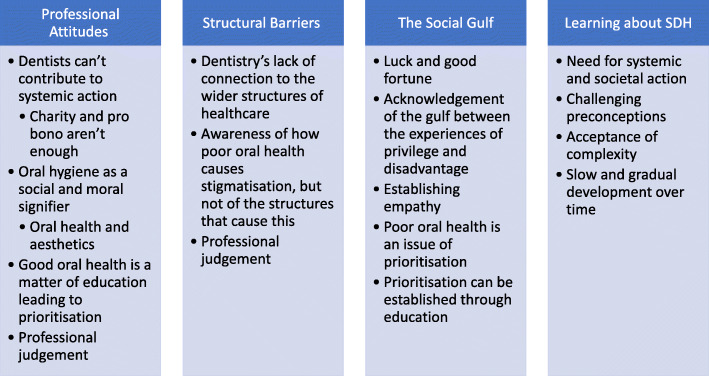
The core themes from this research which demonstrated the students’ understanding of, and engagement with, the social determinants of health

The analysis was carried out by both researchers (ACLH and DL), taking the approach of thematic analysis as described by Braun and Clarke [17]. This method of analysing the data was well suited to enabling the researchers to undertake a rich and detailed exploration of the students’ attitudes towards the social determinants of health. Using this analytical approach involved both researchers cyclically reading and re-reading the students’ reflections, identifying key themes within the texts, examining the patterns in the data that emerged through this process [18]. The researchers compared their coding and examined emerging themes that each other had identified. Several iterations of coding were conducted in this manner until no new themes emerged, and the identified themes could not be reduced or amalgamated further. This process of developing the themes helped to both impart order into the data, as well as cultivated meaning [19]. While no disagreements in coding arose between the researchers, had any eventuated, an impartial third researcher external to this study would have been consulted. The researchers’ interpretation of the data were guided by their respective areas of focus and training; ACLH is a dentally-trained ethicist with specialist-training in dental public health and DL is an academic dentist with a specific focus on educational practice and theory in dental education. Figure 1 illustrates the coding framework developed in the process of analysis.

## Results

Of the 92 students that completed the reflection task, around 45 % were international, with the majority originating from Canada. The remainder of the students were domestic students from Australia. Thirty-two of the 92 students provided consent for their reflective accounts to be included within this research. Due to the de-identified nature of the recruitment and consent process, data were not collected about the demographics of the consenting students.

Following coding and analysis, four inter-related themes emerged: Professional attitudes; Structural barriers; The social gulf, and; Learning about the social determinants of health. These themes are presented below and demonstrated by selected examples of statements written by dental students in their reflection papers.

### Professional attitudes

Professional attitudes towards oral health and the social determinants of health are an important structural factor in how those affected by disadvantage are able to access oral healthcare. This theme collects attitudes towards oral health and dentistry from the students’ reflections that demonstrate their nascent professional perspectives and attitudes towards providing dentistry for those from positions of socioeconomic disadvantage.

Several students expressed in their reflections that the financial barriers to dentistry are unfortunate, although they did not demonstrate any sense of outrage towards this structural barrier to health, with one student seeming to yield to this potential source of injustice:

Often times the patient decides to pull the tooth, not because he or she wants too, but simply because the procedure is too expensive. Though these situations are heartbreaking, the harsh reality is that staying healthy is expensive. – Student 3.

This comment would suggest an acceptance of this status quo, that while harsh, there is nothing particularly objectional to this fact. Another student noted that:

Oral health can be prohibitively expensive, not always for good reasons. – Student 5.

This statement suggests that there are good reasons for dentistry to be prohibitively expensive. One student referenced the experience of one of the guest speakers from ‘The Big Issue’, who had been fortunate in being the recipient of free treatment from a charitable dentist:

The individual had poor diet, exposed to large risks for many systemic diseases and had very poor access to oral health and was lucky enough to be helped by a generous dentist. – Student 30.

While it is undeniable that dentists demonstrating charity towards the public is laudable, there was a lack of outrage that individuals must resort to such measures, rather than oral health being structurally supported through government-funded healthcare. Another student showed more awareness that reliance on the generosity of the dental profession was not a sustainable and realistic approach to managing oral health injustice:

Some dentists even do “pro-bono” work at their own discretion for individual patients. Whilst these are very admirable gestures, unfortunately, these occasional events are not enough to meet the total needs of the community. – Student 32.

These students’ reflections demonstrate a sense of powerlessness to be able to affect and influence structural inequalities as a dentist. It is important that curricula address such sentiments to avoid disillusionment and eventual disengagement.

One student demonstrated that they did appreciate the futility of dentists prioritising professional values:

It is no use droning on about the modified bass technique if your patient is busy calculating if they can afford to eat tonight. – Student 25.

This statement shows an appreciation of the dichotomy between patient and practitioner values is essential if the structural barriers that might prevent a patient from engaging in positive oral health behaviours are to be understood. Many of the student reflections showed that this ability was not yet developed in many of the participants.

Some of the students were able to take part in voluntary community engagement activities external to the curriculum. One participant spoke about their experience speaking with homeless people about oral health at an event where students were invited to provide oral health advice:

I spent a lot of time chatting with people while they waited in line…I felt that chatting in this way was a good use of my time as a lot of people seem to be intimidated by dentists and this kind of communication helps to break down barriers. I loved it when people concluded that I was “far too nice to want to be a dentist” because it made me feel like I had helped to begin breaking down the stereotype of the sadistic dentist. – Student 25.

This participant’s reflection of enjoying breaking the stereotype of the dentist as being unapproachable was shared by another student, who recognised that the power differential between dentists and their patients had the potential to inhibit the clinical relationship:

I noticed they felt that asking such questions was wasting their dentist’s time. They felt intimidated by dentists. I also noticed the same trend at my workplace. When the dentist came over to do a check-up, the patients would not ask many questions but once he left the room, they had so many questions for me. – Student 27.

In reference to the role of professional bodies in the management of the social determinants of health, one student wrote:

The policies created by the ADA [Australian Dental Association] that govern our practice are actually positioned to protect the profession more so than the public. Whilst I feel as though I have heard of this before, I generally brushed this suggestion aside…It was not until we actually started looking at the policies during class that I truly got a glimpse of this subtle bias in the laws and realised how wrong I was. – Student 32.

This student’s understanding of how the dental profession may collectively act to enshrine its own interests above taking positive action to mitigate social determinants that contribute to poor health alludes to an appreciation of how the profession may act as a structural barrier to oral health.

### Structural barriers

This theme examines how the students’ reflections considered and conceptualised structural barriers to health and wellbeing. A large component of this theme relates to a recognition of how poor oral health has the potential to be stigmatising, with clear associations to deprivation and poverty:

Due to the intrinsic structure of our society access to health care is tipped heavily in the favour of those with power and money… There can also be a sense of stigma towards patients with less money and knowledge. – Student 4.

One student demonstrated particular appreciation for the stigmatising impacts of poverty and disadvantage:

There is a man who begs for money near the traffic lights at Sydney Dental Hospital. He has the same backpack as me and he always hides his face… I wonder if the man who shares my style of backpack would have a better prognosis if someone offered him employment? Would he show his face? – Student 25.

Another student spoke of their own embarrassment and shame that, upon entering the dental course, he was exposed to the professional culture of dentistry, where oral and general health are placed in positions of primacy, prompting his reflection upon his prior attitudes towards health:

Whereas in my community, people most often only go when something is hurting them. Hence upon first encountering these views of oral and overall health, I personally felt taken back and to be honest quite embarrassed that I had not held my general health but mainly my oral health to a higher standard. – Student 22.

This comment demonstrates the stigmatising impact that professional judgement may have; in this case, the student judged themselves by the ‘new’ professional standard that participating in the course had exposed them to.

One of the main objectives of the curricula when engaging with the social aspects of dentistry is to expose students to the truth that health outcomes are impacted by structures and elements of society that are distal to the immediate and traditional considerations of health. One student embodied appreciation for this structural reality in their reflection:

Population Oral Health and Professional Practice workshops have definitely taught me to be more critical of the politics around dentistry and has made me more curious (and wary) about the dilemmas I may have to face once I graduate. – Student 32.

We have seen in previous themes that some students felt a degree of inability to affect broader change in the face of structural barriers to health. One student did recognise that they could still play a part in addressing inequity, albeit at a more individual level:

One dental student may not be able to change society or healthcare systems, but I can at least improve the experience for patients on an individual level by having a broader understanding of and compassion for their social circumstance. – Student 2.

The ability to appreciate the impacts of intersectionality on oral and general health is also important for students to develop an appreciation of the structural barriers to oral health:

I also noticed that if someone was suffering from one obvious adverse social determinant such as homelessness, they were often suffering from many adverse determinants such as low education, social isolation, and aboriginal status. This “clustering” of adverse factors makes it even harder for these individuals to receive and maintain the care from health care practitioners…[the guest speaker] had a number of common adverse social determinants of health manifesting together. – Student 29.

This appreciation for how determinants of health may interact and amplify the impacts of deprivation is important. This understanding of the synergistic effects of structural and social determinants of health is helpful in assisting students to overcome the social gulf between their own experiences and those of the most disadvantaged who carry the highest burden of disease.

### The Social Gulf

This next theme considers the social gulf that is in evidence between the students’ experiences of hardship and deprivation, and of those who experience poverty and the conspiracy of other social determinants of health against their wellbeing. Speaking on this phenomenon, Mary Otto stated; “The social gulf between dentists and poor patients is manifested in ways that may compound the challenges of delivering care. It can be hard for better-off people to understand the barriers to poor face in accessing health care services.” [20] This theme explores how the students’ reflections expose how the social gulf manifests itself in reference to how the students conceptualise the social determinants of health.

Being exposed to the perspectives of guest speakers who had experienced homelessness and deprivation first-hand helped students to gain insight into how the structure of the healthcare system may be a serious impediment for the most disadvantaged in society, in a way that differed starkly from their own realities:

The chief takeaway point I gathered…was how much [the speaker’s] perspective of the healthcare system differed from mine: I do not feel like I will be negatively judged in any way when visiting healthcare services or applying for low-income benefits to assist with healthcare. It is a much different experience for someone who is unable to provide residency details for the paperwork required…Not only does the bureaucratic process immediately become more drawn-out and complicated, the homeless person is also constantly being talked down to or viewed in disgust for perceived dirtiness. Yet, it is precisely these people with poor hygiene who usually require treatment the most, as their current state is a direct result of their social circumstance. – Student 2.

While my oral health was always a priority, it has come to my attention that unfortunately not everyone has access to oral healthcare to make it as much of a priority as I do. DMD1 has definitely been an eye opener to me in terms of how much I have been taking my ease of access to oral healthcare for granted. – Student 14.

Another student wrote about her similar reflections on structural inequalities following an experience of taking part in a volunteering opportunity offered by the school, where they spent time conversing with homeless clients at an event offering various services, including basic oral health assessments which the students were participating in:

Many individuals that I talked with that day explained to me their experience of navigating the health system to receive dental care. They spoke of extended waiting times, difficulty travelling to the clinic/hospital, distrust of the system and frustration at the complexities of paperwork among other things. Some of these individuals had resultingly not seen a dentist in years despite being aware, hoping to improve and sometimes self-conscious of, issues developing in their mouths. – Student 15.

In these reflections, these students demonstrate that they have developed awareness of the gulf between their own experiences and those of individuals impacted by homelessness and deprivation. Their reflections also demonstrate a clear development of awareness of the structural barriers to oral health.

One student reflected upon how their own background and upbringing had led them to assume that their experience of orthodontic treatment was common to all within society, rather than being an expensive treatment that frequently has connotations with privilege and status:

All the people around me had straight teeth; it was almost a rite of passage. However, I did not realize until later that this was not the reality for most of the population. I remember one of my first girlfriends had a crooked lower arch. I remember asking her in the most naïve (and borderline ignorant) way, “You didn’t have braces growing up?” – Student 31.

Many of the students recounted in their reflections that their experiences in the first year of the course, in particular the guest presentation from the team at the Big Issue, had challenged their preconceptions around the causes of poor oral health. Students recalled how they had previously associated poor oral health with conscious life-choices tantamount to laziness and deliberate self-neglect; perspectives which they had now grown to question:

Before commencing the DMD program this summer (Jan 2018), I had a very naïve view on the factors that influence oral health. I believed that oral health mainly depended on a patient’s “motivation for nice teeth”. I felt that someone with poor oral health either had poor discipline to regularly brush and floss their teeth, had low will-power to resist sugary treats, or had little self-care for themselves. – Student 10.

I will always remember the special guest presentation about homelessness. It was such an eye-opening presentation because I learned so much about the struggles of homelessness which involved being treated unfairly, misjudged and abused. Often homelessness is due to mental disorders, physical disabilities, being laid off from a job and family problems rather than because that person is lazy. – Student 11.

Earlier this year we had a talk from homeless or ex-homeless people from the Big Issue and this was a huge eye opener for a sheltered petal like myself. My previous thoughts consisted of ‘Brushing teeth takes about 3 minutes just do it, it’s not that hard. Maybe I can excuse lack of flossing because it’s annoying.’ And in all honesty I am quite ashamed that I could ever think of this in such a black and white way…have been humbled in realising that yes, for someone like me it is 3 min of my day and not hard to complete in my dull routine. – Student 16.

[The guest speaker] mentioned that while she was homeless she was very aware her oral hygiene wasn’t the best, yet she simply didn’t have a choice because she was focused on trying to find shelter day to day. This experience helped me learn that as a prospective dentist I need to have a wider perspective when treating patients and observing stranger’s oral hygiene, which includes not being judgemental and realising that not maintaining oral hygiene isn’t necessarily a choice for everyone. – Student 12.

The social gulf was particularly apparent in one student’s reflection, where they suggested that their experience of being out of part-time work for two weeks, meaning that they needed to cancel their gym membership, meant that they had experience of deprivation:

Hence, from first-hand experience, I can understand how lower economic statuses may result in an individual choosing more sugary, fattening, and unhealthy dietary items that my lead to decreased oral health, increased carious lesions, and a generalized decline in their overall systemic health. – Student 9.

While it may be laudable for students to engage in exercises of empathy in order to gain better understanding of the experiences of those suffering deprivation, there is also a risk that the enormity of the social gulf in this instance may lead to an erosion of sensitivity. There is a stark contrast between acute hardship that is quickly overcome, and systematic deprivation that is almost impossible to escape. One student stated that:

Not everyone values their oral aesthetics and similarly, not everyone values their oral health. As future dentists it is imperative to recognize this to avoid the paternalistic attitude that often interferes with delivering the most personalized and effective care. – Student 31.

This statement demonstrates an assumption that an inability to prioritise oral health is equivalent to a lack of value being placed in oral health, also conflating dental aesthetics with the entirety of oral health.

### Learning about the Social Determinants of Health

The students’ reflections provided a valuable insight into how students begin to learn and conceptualise the relevance and management of the social determinants of health to dentistry. This theme explores how the students reported that their educational experiences in the first year of their course had developed their understanding in the context of professional attitudes, structural barriers and the social gulf.

Learning about complex phenomena such as the social determinants of health and their impact on health and oral health is longitudinal and progresses from less sophisticated understandings to more developed understandings as learners make small turns from their current stances [21]. Overwhelmingly, the students reported a starting or current belief that the social determinants of health required management through patient education, and that low socioeconomic status was the cause of a lack of understanding of the effects of particular lifestyle choices and behaviours. The students expressed a belief that through chairside education, these patients might be aided to reflect upon their circumstances and affect positive change:

I believe that this negative chain of dominoes can be broken with the right information and attitude towards my future patients… And most often it is not because the people are not smart enough to understand, but more that they have not been properly informed. – Student 3.

The highest priority for every health professional should be education. Educating your patients (whether through layman’s terms or medical terms) will encourage them to maintain not only better oral health, but an overall healthier lifestyle. – Student 5.

being better educated not only provides knowledge about health conditions, but also acts as a motivating factor (which may in fact stem from fear of deterioration of our health condition). – Student 7.

One student noted that, for them, the improvement of oral hygiene in themselves through studying the course was a motivating factor in educating others:

Overall, my oral hygiene has definitely improved significantly over this first year in the DMD program, and I definitely find myself prioritizing on educating others on the importance of oral hygiene not just for myself, but I think it is beneficial to everyone. – Student 14.

One student, whilst expressing similar perceptions that education was key to the management of the social determinants of health, also recognised that change was not simply a matter of choice:

We recognise the determinants of health because we are studying them, but we need to be careful not to assume everyone else knows or understand them. We need to appreciate that not everyone has had the opportunity to learn about health and as such should not be judged because of poor choices they may assume to be fine or correct. It’s also not enough just to understand the choices you are making, but you must also have the means to enact the change. - Student 1.

Despite the curriculum addressing the often-illusory nature of choice for those from disadvantaged socioeconomic groups, some students demonstrated in several instances a reliance on the notion of choice in their conceptualisations of the social determinants of health:

Everyone deserves equal opportunity to make choices that lead to good health. – Student 8.

This apparent juxtaposition of positions could represent the conceptual difficulty and longitudinal nature of learning about the social determinants of health. As a beginner in a new academic area, students lack the specialised conceptual language to express their position, thus clashing ideas flow into others. Similarly, complexity reduction and deterministic thinking was evident as there was a tendency to suggest that a lack of education led to a lack of interest or care in good oral or general health:

Social groups with less access to proper education regarding general and oral health are often unaware of the effects of their lifestyle habits, or unaware of the significance of oral health (i.e. don’t know enough to care). – Student 24.

One student suggested that while they had received instruction on the nature of the social determinants of health during previous courses, they had not considered the relevance of this to oral health:

I had a basic/theoretical understanding of social determinants of general health through my undergraduate degree (pharmacy), however I had never thought of the impacts it would have on one’s oral health up until I started dentistry. – Student 19.

According to Boler’s pedagogy of discomfort [22], discomfort can be a provocation to think differently about something, thus discomfort can provide a stimulus to consider other points of view. The discomfort felt when challenged by images of people in different circumstances to their own was evident:

Homeless people in the streets of central Sydney were a confronting sight when I first moved here, and the guest visitor early in the year provided even more difficult insight to the homeless situation. – Student 2.

The value of directly exposing students to the narratives of those who have lived and authentic experience of poor health due to deprivation was further encapsulated by one student who expressed how the learning sessions had impact upon them:

I often think of [the guest speaker] from the Big Issue who came to speak to us in first semester… I am so grateful that we had the opportunity to listen to [her] story – it was an experience that will stay with me forever. – Student 25.

Providing students with the opportunity to learn from immersive experiences would appear to be important in providing conditions for students to build a meaningful conception. While the first-year curriculum clearly has not provided a holistic understanding of the social determinants of health to all students, helping students to begin the journey of greater empathy and understanding of what is clearly an alien perspective for many, is essential. Developing a future oral health workforce that is able to respond to the needs of the most vulnerable in society should be a key objective in dental education.

## Discussion

The students who permitted the use of their reflections in this analysis were all at the beginning of their journey through dental education at the time that they took part in this assessment task. There is an understandable variation between the student participants regarding the depth and extent to which their respective reflections demonstrate understanding of the social and structural determinants of health. The aspiration is that through further didactic and experiential learning, all students would be able to demonstrate structural competency with regards to understanding the social determinants of health before they graduated and entered the oral health workforce.

Metzl and Hanson [16] provide five core competencies to their notion of structural competency: (1) recognising the structures that shape clinical interactions; (2) developing an extra-clinical language of structure; (3) rearticulating “cultural” formulations in structural terms; (4) observing and imagining structural interventions; and (5) developing structural humility. The themes described within the results will be further considered within the context of these five competencies.

### Recognition of structures that shape clinical interactions

Metzl and Hansen argued that considering this competency is a productive way for students to begin to understand the upstream influences on healthcare interactions, for example, how policies surrounding insurance or publicly-funded care might impact the patient:dentist interaction. Of particular relevance to this competency is the ability for students to appreciate how a patient’s ability to pay for dental care is likely to ultimately affect their oral health outcomes. It is evident within the students’ reflections that some students are acutely aware of this barrier to oral health and access to services. However, there is also evidence from some students that the financial cost of dental treatment is an ‘acceptable’ barrier, one that does not elicit or justify outrage or reform. In expressing their powerlessness to effect change, many students proposed responses that showed that were determined to find solutions on an individual patient level. Apelian et al. [23] highlight that the emphasis on dentistry as a craft, with an artistic vision places high importance on surgical and high-technology interventions and less on understanding patient perspectives. By taking account of the collective professional identity of dentists, students may be encouraged to question these taken-for-granted assumptions about the profession they are entering.

Some students also mentioned how their experiences listening to and interacting with the Big Issue guest speaker helped them to understand how some patients were impacted by institutional policies and approaches when they were seeking care, for example, not having the right documentation or suffering stigmatisation from health service staff. Prior research examining the experiences of patients seeking publicly-funded at dental schools found that structural barriers such as being made to wait in a separate line in the waiting room to privately-paying patients and receiving different treatment from clinic staff, was stigmatising [24]. Our students, the future of our profession, have their professional nascence in such environments; the hidden curriculum is a powerful informant of developing professional attitudes and behaviour [25].

### Developing an extra‐clinical language of structure

One of the authors (ACLH), remembers starting his first role teaching ethics in dental academia, being counselled by a senior and prominent colleague within the school; “Remember, the students are here to be dentists, not ethicists”. Whilst the day-to-day technical skills of being a dental practitioner are essential components of dental curricula, students need to be able to situate these skills in the wide social and cultural milieu in which they live and work. Complex concepts linked to the social determinants of health cannot be understood using only the framework provided by biomedicine; where the biological impacts of social structures are well understood, but the nature of social structures themselves are relatively unexplored. Relying purely on a dental framing to learning about the social and structural determinants of health is therefore unhelpful. In the students’ reflections, we encounter a lack of extra-clinical conceptualisation of the social determinants of health. Most students considered their role in addressing the social determinants of health through the delivery of clinical oral hygiene education. The juxtaposition of ideas in student reflections could signal that when learning a new and complex language, expression of ideas may be inarticulate. Thus, developing an extra-clinical language of structure forces educators to sustain attention towards the social determinants of health such that the learners experience the variation needed to form more sophisticated and actionable understandings.

The notion of inclusion of health posed by Freeman et al. [26] is consistent with the findings of our research, their definition being: “Inclusion oral health is based on a theoretically engaged understanding of how social exclusion is produced and experienced, and how forms of exclusion and discrimination intersect to compound oral health outcomes. Inclusion oral health focuses on developing innovative inter-sectoral solutions to tackle the inequities of people enduring extreme oral health.” Within our work, we found evidence of students exhibiting judgemental attitudes and a lack of understanding towards those impacted by deprivation. In order to help students to appreciate the inter-sectoral nature of concepts such as inclusion oral health, greater collaboration within the curriculum with other health professional students in inter-professional learning should be explored and utilised where appropriate.

The hidden curriculum is a major barrier to dental students meaningfully learning about the social determinants of health – students learn professional attitudes quickly, and many even enter dental school with a familial background in dentistry. The nature of dental education, dominated by clinical disciplines, means that students learn general practice dentistry in the specialist compartments. Each of these disciplines has its own ways of thinking and practising, and the language does not necessarily include reference to the social determinants of health. Thus, integration of the social determinants of health into clinical practice structurally is a big challenge.

### Rearticulating “cultural” formulations in structural terms

Within the five competencies, this competency is concerned with the development of students’ abilities to discuss cultural determinants of health in the context of the deeper, complex cultural structures that lead to health inequalities, rather than attributing the social determinants of health to cultural factors. In the Australian context, a key example of how opportunity to develop this competency might be missed would be using the example of an Indigenous Australian in a clinical case, considering surface cultural factors (such as rural and remote geographic location), whilst not considering the approximately 200 years of deprivation, persecution and stigmatisation that First Nations Peoples have faced in Australia since colonisation.

In the students’ reflections, only one student made reference to Aboriginal and Torres Strait Islander status as having relevance to the social determinants of health. It may be that the first year DMD students were not currently considering cultural structures and their contribution to the social determinants of health and health outcomes.

### Observing and imagining structural intervention

Clearly structural barriers to health are not immovable objects; each structure that impacts health and illness are the product of some policy, financial or cultural decision that has been made at some point in time. Students should be encouraged to consider how these structures might be subject to some form of intervention.

Some students demonstrated an awareness of the political structures surrounding oral health, appreciating that these required to profession to engage in higher-level advocacy to address structural inequalities. One student specifically recognised the part of the professional association in maintained structural barriers to health, through self-interested policies [3].

Most students showed a lack of appreciation for the complexities of social structures, their role in creating and maintaining inequality and how dentists might intervene against structural barriers to health. Many stated that they saw their role as educators, but several students also noted the limitations of education as a sole approach to addressing structural barriers. While the first-year course in DMD may sensitise students to the social determinants of health, many students appear to have been unaware of how they might contribute to their amelioration.

### Developing structural humility

Structural humility calls for students and qualified professionals to strive to address structural inequalities, while also realising the limits and boundaries of medicine in addressing these barriers to health. Sharma et al. [27] point out that education about the social determinants of health does not necessarily result in reduction of inequities, and that curricula that are rich in content but low in actions may not provide optimum conditions for student learning. Thus, systemic change on the social determinants of health is typically slow and non-uniform, and students can learn that the social determinants of health is a multifaceted, complex phenomenon that the health professions cannot solve on their own. While many students proposed simplistic solutions, there was also a sense that this issue is bigger than one that could be solved by a single practitioner or profession. Transdisciplinary research approaches can be adopted to generate new framings and analyses of an issue to grasp the issue’s complexity. This is achieved by considering diverse perspectives and integrating abstract and case-specific knowledge to produce practical knowledge to address the issue [28]. Dulin et al. [29] demonstrated a trans-disciplinary approach in using geospatial modelling and community-based participation in order to ameliorate health disparities through enhancing access to primary health services. Dental education must similarly engage with the principles of inclusive community participation and inter-disciplinary learning in order to teach that addressing oral health disparities requires the decolonisation of oral health and a broader engagement with key stakeholders.

## Trustworthiness and limitations of study:

We used the trustworthiness measures as defined by Lincoln and Guba [30] to guide the rigor of the study. As data collected was written by the participants, data can be considered *credible*. While these findings do not claim to be generalisable, we do invite dental educators to consider the *transferability* of the study as it relates to their own particular context. Describing the steps taken in the study and providing extracts from participant’s written reflections, the *dependability* and *confirmability* of the study is displayed to readers. Our awareness of the mutual relationship between us as researchers and the object of study drew continual *reflexive* attention to the researchers as part of the world being studied. The question of how to represent the participant voices is central to reflexivity and we saw it as an intentional activity where we could challenge our values and assumptions in the research setting.

Limitations of this study relate to the single research site and single cohort of students involved in the research. Future research studies that extend this work could track students longitudinally as they progress through dental school, or involve more research sites.

## Conclusions

In this exploration of first year postgraduate dental students’ reflections, we have demonstrated the chasm that exists between the experiences of the student body and those impacted most severely by the social determinants of health. This social gulf between dental students and people who are unlike themselves creates conditions for misunderstanding, attribution (deterministic thinking) and judgment. From the narratives within the reflections, students may quickly adopt professional attitudes that link oral hygiene practices and patterns of attendance to social status, heightening the stigmatisation associated with experiencing poor oral health.

Learning about the social determinants of health is not easy and requires sustained, longitudinal attention in dental school – the first-year experiences show some shifts in some students, with more integrated learning experiences needed. Awareness of the hidden curriculum and continued education of the educators across all curriculum areas is needed if education on the social determinants of health will work. The ability of the graduates of dental programs to be able to offer inclusive oral health care is an essential competency and therefore needs to have prominence as a whole-of-curriculum approach.

## Data Availability

The data that support the findings of this study are fully presented within this work.
